# Prevalence and healthcare burden of pulmonary alveolar proteinosis

**DOI:** 10.1186/s13023-018-0846-y

**Published:** 2018-07-31

**Authors:** Cormac McCarthy, Ruzan Avetisyan, Brenna C. Carey, Claudia Chalk, Bruce C. Trapnell

**Affiliations:** 1 Translational Pulmonary Science Center, Children’s Hospital Medical Center, Cincinnati, OH USA; 2 Sanofi, Cambridge, MA USA; 3 Division of Pulmonary, Critical Care, and Sleep Medicine, University of Cincinnati College of Medicine, Cincinnati, OH USA; 4 PAP Foundation, Denver, CO USA; 5 Division of Pulmonary Biology, Cincinnati Children’s Hospital Medical Center, 3333 Burnet Avenue, Cincinnati, OH 45229-3039 USA

**Keywords:** Pulmonary alveolar proteinosis, Prevalence, Healthcare burden, GM-CSF autoantibody

## Abstract

Pulmonary alveolar proteinosis (PAP) is a rare syndrome of alveolar surfactant accumulation, resulting hypoxemic respiratory failure, and increased infection risk. Despite advances in our understanding of disease pathogenesis and the availability of improved diagnostics, the epidemiology and healthcare burden of PAP remain poorly defined. To determine the prevalence, and healthcare utilization and costs associated with PAP, we interrogated a large health insurance claims database containing comprehensive data for approximately 15 million patients in the United States. We also evaluated data from a referral-based diagnostic testing program collected over a 15-year period. The prevalence of PAP was determined to be 6.87 ± 0.33 per million in the general population, similar in males and females, and increased with age, however considering difficulties and delays in diagnosing this is likely a minimum estimate of true prevalence. PAP patients had significantly more comorbidities, health care utilization and associated costs compared to control patients precisely matched for age and gender. Between 2004 and 2018, 249 patients confirmed to have PAP were evaluated to identify the PAP-causing disease; 91.5% had autoimmune PAP, 3% had hereditary PAP caused by GM-CSF receptor mutations, 4% had secondary PAP, and 1.5% had congenital PAP. Considering the high diagnostic accuracy of serum GM-CSF autoantibody testing and predominance of autoimmune PAP, these results emphasize the importance of utilizing blood-based testing in PAP syndrome to identify the PAP-causing disease rather than invasive lung biopsies, resulting in earlier diagnosis, reduced morbidity and lower healthcare costs.

## Introduction

Pulmonary alveolar proteinosis is a rare syndrome characterized by progressive alveolar surfactant accumulation and hypoxemic respiratory failure that occurs in various diseases commonly categorized as primary, secondary or congenital PAP [1, 2]. Primary PAP accounts for the majority of cases and is caused by disruption of granulocyte-macrophage colony-stimulating factor (GM-CSF) signaling, either by GM-CSF autoantibodies (i.e., autoimmune PAP) [3] or genetic mutations involving the GM-CSF receptor (i.e., hereditary PAP) [4]. Secondary PAP occurs in a heterogeneous group of conditions that reduce numbers or functions of alveolar macrophages and thereby surfactant clearance. Congenital PAP is caused by mutations in genes required for normal surfactant production [1, 2]. Despite pathogenic advances and improved diagnostics, the prevalence and healthcare burden of PAP remain poorly-defined: formal epidemiologic studies have not been previously reported. We evaluated the epidemiology and healthcare burden of PAP based on data from a large health insurance claims database and a referral-based PAP diagnostic testing program.

## Methods

Study data were derived from the OptumInsight database [5], a repository of de-identified, Health Insurance Portability and Accountability Act-compliant data for 30 million unique healthcare-insured members (patients) at the time of the study from across the United States (US); tracked longitudinally to comprehensively capture data from inpatient, outpatient, emergency, and pharmacy-related health insurance claims (claims data). Patients were included in this study only if they had complete annual claims data in two or more consecutive years. Included patients were designated as having PAP in a study year if they had at least one claim containing an international classification of diseases (ICD)-9 code of 516.0 (the diagnostic code for PAP) in that year. Annual PAP prevalence was defined as the number of PAP patients identified divided by the total number of included patients in the same study year.

Relative healthcare utilization and costs were determined using a case-control approach. Cases comprised included patients with at least one ICD-9516.0-associated claim and controls were included patients without any ICD-9516.0 code-associated claims. Cases and controls were precisely matched one-to-one for age and gender. Only years with complete annual claims data were included in the analysis. Demographics, comorbidities, and annual per-patient healthcare utilization and costs were calculated for cases and controls and compared.

As part of a concurrent separate study, an independent cohort of patients were evaluated to determine the underlying cause of PAP syndrome. Patients were diagnosed with autoimmune PAP by the use of a serum GM-CSF autoantibody test with confirmation by the STAT5-phosphorylation index test to demonstrate inhibition of GM-CSF signaling [6, 7] while other PAP-causing diseases were diagnosed by DNA sequencing, receptor analysis, and other methods under an institutional review board-approved protocol at the Translational Pulmonary Science Center in Cincinnati. Numeric data are presented as mean ± SEM. Statistical analysis was performed using Prism 7.0 or SAS 12.3.

## Results

**Fig. 1:**
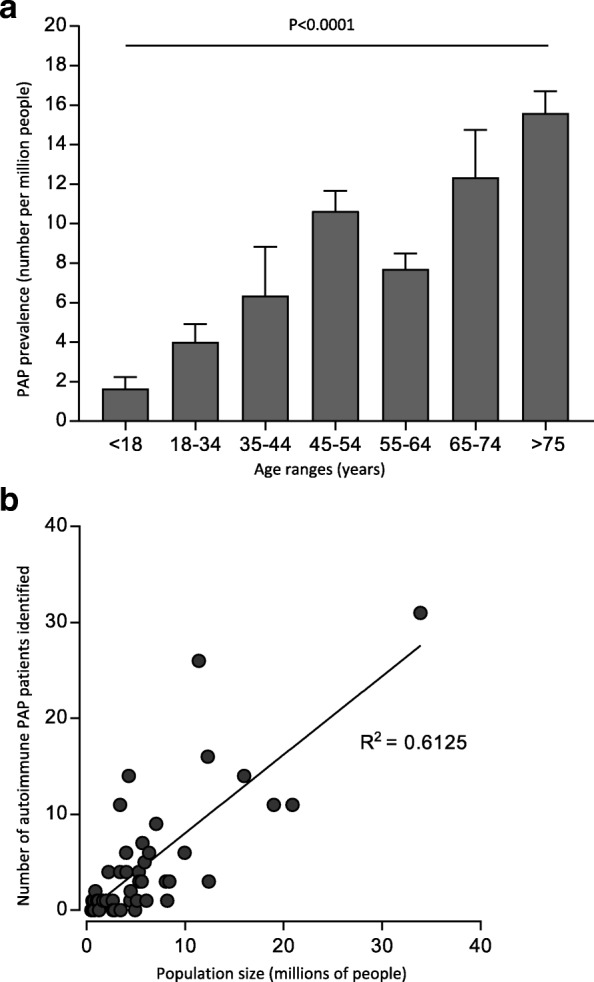
Prevalence of PAP syndrome and detection of autoimmune PAP in the United States (US). **a** Annual prevalence of PAP syndrome was determined retrospectively between January 1, 2008 and December 31, 2012 using data from the OptumInsight health insurance claims database. Bars represent the mean (±SD) prevalence stratified by age (left) or gender (right). Statistical comparisons were done with ANOVA or Student’s t-test, respectively. **b** Relationship between detection of autoimmune PAP and population size. Autoimmune PAP was identified among individuals with PAP syndrome across the US between 2004 and 2018 by serum GM-CSF autoantibody testing [6] with confirmation by blood-based STAT5 phosphorylation index testing to document impaired GM-CSF signaling [7] at the Translational Pulmonary Science Center in Cincinnati. Data are expressed as the number of individuals with autoimmune PAP by state plotted against the state population obtained from the 2010 US Census [8]. The correlation between GM-CSF autoantibody positive PAP patients and state population size was evaluated by Spearman correlation

The study population included 15,011,522 ± 175,519 patients (8,144,741 ± 84,094 females and 7,766,782 ± 91,560 males) annually between 1/1/2008 and 12/31/2012 comprising 5.16 ± 0.04% of the US population. PAP prevalence increased with age in bimodal distribution (Fig. 1a) but did not vary with gender (Fig. 1a, Table 1). The annual prevalence of PAP in the general population was 6.87 ± 0.33 per million (Table 1). Using this value and 308.7 million for the US population size [8], the number of PAP patients in the US was estimated to be 2120.

**Table 1:** Study Population and Annual Prevalence of PAP Syndrome in the US^a^

Study year	Total patients^b^	Total PAP patients^c^	Total PAP prevalence per million patients^d^
2008	15,519,985	105	6.77
2009	15,686,604	127	8.10
2010	16,070,700	103	6.41
2011	15,764,422	109	6.91
2012	16,515,902	102	6.18
Mean	15,911,523	109	6.87
Study year	Total female patients^b^	Female PAP patients^c^	PAP prevalence per million female patients^e^
2008	7,953,672	51	6.41
2009	8,038,763	67	8.33
2010	8,233,635	57	6.92
2011	8,069,748	67	8.30
2012	8,427,886	59	7.00
Mean	8,144,741	60	7.39
Study year	Total male patients^b^	Male PAP patients^c^	PAP prevalence per million male patients^f^
2008	7,566,313	54	7.14
2009	7,647,841	60	7.85
2010	7,837,065	46	5.87
2011	7,694,674	42	5.46
2012	8,088,016	43	5.32
Mean	7,766,782	49	6.31

*Definition of abbreviations*: *PAP* pulmonary alveolar proteinosis

^a^Patients are individuals represented by de-identified data in the OptumInsight database during the study period meeting the inclusion and exclusion criteria as defined in the methods

^b^Includes all (or female/male patients as indicated) with complete annual claims data for at least two consecutive years

^c^Includes all (or female/male patients as indicated) patients with at least one claim associated with an ICD-9516.0 code in the respective calendar year

^d^Calculated as the number of PAP patients in each year divided by the total number of patients included in that year

^e^Calculated as the number of female PAP patients in each year divided by the number of female patients included in that year

^f^Calculated as the number of male PAP patients in each year divided by the number of male patients included in that year

Comorbidities were higher in PAP than control patients as determined by the Charlson Comorbidity Index (CCI) [9] adapted for ICD-9-coded databases [10]. The CCI for PAP and control groups were 1.84 ± 2.48 and 0.55 ± 1.44, respectively (*p* < 0.0001) (Table 2). PAP patients had increased healthcare utilization and costs compared to controls attributable, respectively, to increased outpatient visits (17.30 ± 13.77 versus 10.40 ± 11.38; *p* < 0.01), emergency room visits (1.49 ± 1.17 versus 1.08 ± 0.27; *p* = 0.014), and longer hospital stays (15.96 ± 20.71 versus 5.40 ± 5.07 inpatient days; *p* = 0.027). The annual per-patient healthcare costs were 5-fold higher among PAP patients than controls ($54,865 ± 95,524 versus $10,214 ± 20,233, respectively; *p* < 0.001). This difference was due to increased costs of inpatient visits (2.7-fold, *p* = 0.04), outpatient visits (3.8-fold, *p* < 0.001), and prescriptions (4.75-fold, *p* < 0.001), but not differences in emergency room visit costs (*p* = 0.563).

**Table 2:** Charlson Comorbidity Indexed Conditions in PAP Syndrome compared to control individuals

Indexed Comorbidity	PAP	Control	*p* value
Myocardial Infarction	9 (5.5%)	4 (2.4%)	0.157
Congestive Heart Failure	30 (18.3%)	5 (3%)	< 0.001
Peripheral Vascular Disease	10 (6.1%)	4 (2.4%)	0.101
Cerebrovascular Disease	15 (9.1%)	4 (2.4%)	0.009
Dementia	0 (0%)	1 (0.6%)	0.317
Chronic Pulmonary Disease	71 (43.3%)	16 (9.8%)	< 0.001
Connective Tissue Disease	14 (8.5%)	1 (0.6%)	< 0.001
Peptic Ulcer Disease	3 (1.8%)	2 (1.2%)	0.652
Mild Liver Disease	15 (9.1%)	2 (1.2%)	0.001
Diabetes without Complications	36 (22%)	20 (12.2%)	0.019
Diabetes with End-Organ Damage	8 (4.9%)	2 (1.2%)	0.054
Hemiplegia/Paraplegia	1 (0.6%)	0 (0%)	0.317
Renal Disease	16 (9.8%)	2 (1.2%)	< 0.001
Non-metastatic Cancer Hematologic Malignancy	15 (9.1%)	6 (3.7%)	0.042
Moderate/Severe Liver Disease	2 (1.2%)	0 (0%)	0.0156
Metastatic Solid Tumour	2 (1.2%)	2 (1.2%)	> 0.9999

Between 2004 and 2018, 700 patients from 25 countries including 400 US patients participated in a laboratory-based research protocol to identify and study PAP-causing diseases. Among 249 US patients with confirmed PAP syndrome, 228 (91.5%) had a positive serum GM-CSF autoantibody test identifying autoimmune PAP, 7 (3%) had hereditary PAP caused by mutations in *CSF2RA* or *CSF2RB* (encoding GM-CSF receptor α or β chains), 10 (4%) had secondary PAP, and 4 (1.5%) had congenital PAP. The number of patients with a positive GM-CSF autoantibody test in the US varied by state (e.g., 1 in Vermont - pop. 608,827; 31 in California - pop. 33,871,648) and correlated with population density (Fig. 1b)(R^2^ = 0.6978, *p* < 0.0001).

## Discussion

We determined the prevalence, healthcare utilization and costs of PAP in the US based on analysis of data from 5% of the US population, independent of bias related to age, gender, geographic location, environmental exposures, or ethnicity and PAP pathogenesis based on laboratory testing. Separately, we identied PAP-causing diseases in a cohort of patients referred to our site for tertiary evaluation and diagnosis. To our knowledge, these results comprise the first measurement of PAP prevelance in the US.

We believe our measurement of PAP prevalence in the US should be considered a minimum estimate and may underestimate actual prevalence for several reasons. First, since PAP typically presents as slowly progressive dyspnea of insidiouse onset, patients can remain asymptomatic for long periods before coming to medical attention. In fact, some patients are diagnosed incidentally, for example, when an abdominal CT scan identifies characteristic radiological findings in the lower lung fields. Further, in a report on the Japanese National PAP Registry, a large proportion (69/223, 31%) of PAP patients were asymptomatic and might have been missed if patient identification methods had not included mandatory health screening [11].

Second, symptomatic PAP patients are often misdiagnosed as asthma or pneumonia (before or after chest radiography, respectively) until failure to respond to ‘appropriate’ therapy prompts reconsideration and diagnostic testing. Notwithstanding, the PAP prevalence we determined among individuals of all ages in the US (6.87 per million) is similar to but slightly higher than the prevalence of PAP among adults in Japan (6.2 per million) [11]. Our observation that autoimmune PAP accounts for 91.5% of US PAP patients also agrees well with results from the Japanese National PAP Registry [11].

Our results indicated the prevalence of PAP syndrome increased with age across a broad range of ages including patients over 75 years old. It is important to note that our study did not address the incidence of PAP. Thus, it is possible and, perhaps likely, that the older individuals with PAP may have presented at a younger age.

Based on our minimum estimate of 2120 PAP patients in the US and experience of a relatively small number of referrals in an active diagnostic testing program (400 US patients over a 14-year period), we conclude that testing to diagnose specific diseases causing PAP syndrome is underutilized and that many patients with autoimmune PAP remain undiagnosed. Considering the high diagnostic accuracy of serum GM-CSF autoantibody testing [6] and prevalence of autoimmune PAP, the diagnostic insensitivity and morbidity associated with lung biopsy-based testing, and emerging treatment options for specific PAP-causing diseases, our results underscore the importance of increased use of blood-based testing in PAP patients, which can identify the PAP-causing disease, lead to earlier diagnosis, and reduce test-related morbidity and cost.

## Abbreviations

CCI: Charlson Comorbidity Index
DNA: Deoxyribonucleic acid
GM-CSF: Granulocyte/macrophage colony-stimulating factor
ICD: International Classification of Diseases
PAP: Pulmonary alveolar proteinosis
STAT5: Signal transducer and activator of transcription 5
US: United States
